# Development of underwater radiography scanner for reactor-pool experiment at the TRIGA PUSPATI reactor

**DOI:** 10.1016/j.mex.2018.10.011

**Published:** 2018-10-24

**Authors:** Mohamad Annuar Assadat Husain, Suhairul Hashim, Norasalwa Zakaria, Muhammad Rawi Mohamed Zin

**Affiliations:** a Department of Physics, Faculty of Science, Universiti Teknologi Malaysia, 81310 UTM Skudai, Johor, Malaysia; b Malaysia Nuclear Agency, Bangi 43000 Kajang, Selangor, Malaysia; c Atomic Energy Licensing Board, 43800 Dengkil, Selangor, Malaysia

**Keywords:** Underwater scanner using radiography technique, Scanner, Collimator, Radiography, Pixel, RTP

## Abstract

This paper describes the development of a custom-designed underwater scanner to support the experimental works for characterizing irradiated fuel stored in the TRIGA PUSPATI pool by means of radiography technique. Materials used to build the scanner are aluminum 6061, lead and teflon. Three main units that make up the scanner are rig structure, arm block and collimator. Collimator is designed to control radiation exposure by opening and closing the shutter. The experimental works were conducted underwater at 5-m depth hence water tightness is one of the main design criteria. Radiation in terms of gamma energy is captured by radiography film which after development and processing revealed the image of the radiation impact in terms of pixel and gray value. The film size used is 4in x 8in which was slot in the collimator. To validate the scanner, fuel element containing 8.5 wt% and 12 wt% enriched Uranium 235 were used. It was found that the experimental output is consistent with the fuel type and confirmed that the scanner is viable for fuel characterization study.

## Graphical abstract

**Figure:**
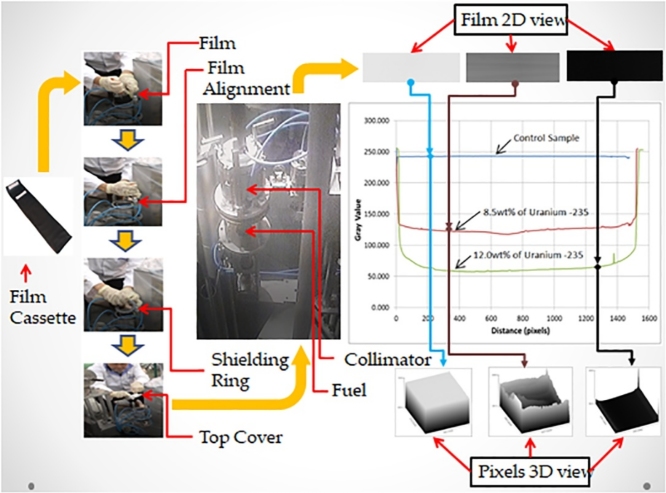

Specifications Table

**Table:** 

Subject Area	• *Engineering* • *Physics and Astronomy* • *Materials Science*
More specific subject area	*To development of a custom-designed underwater scanner to support the experimental works for characterizing irradiated fuel stored in the TRIGA PUSPATI pool using radiography technique.*
Method name	*Underwater scanner using radiography technique*
Name and reference of original method	*NA*
Resource availability	*NA*

## Method details

**Fig. 1:**
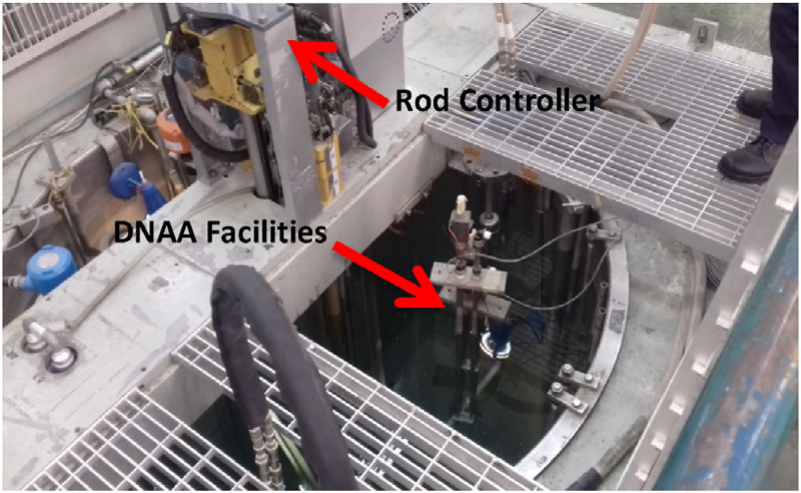
The top view of TRIGA PUSPATI reactor pool.

This study is a first attempt towards conducting an underwater experiment to be carried out in the reasearch reactor TRIGA PUSPATI (RTP). The RTP is a pool type reactor, where the reactor core sits at the bottom of a 6.3 m height lined with aluminum [1]. The reactor uses solid fuel elements in which the zirconium-hydride moderator is homogeneously combined with enriched Uranium-235. Demineralized water acts both as coolant and neutron moderator, while graphite acts as a reflector. The 2 m-diameter reactor pool is surrounded by biological concrete shielding. It houses piping system, storage rack, pneumatic system and other instruments that are installed for Neutron Activation Analysis (NAA), and Delay Neutron Activation Analysis (DNAA). These make the space inside the reactor pool really tight and constrained, and present the key challenge when designing the scanner. Fig. 1 shows the top view of the TRIGA PUSPATI reactor pool before the underwater scanner is installed in the pool.

The objective of this study is to examine the feasibility of underwater gamma scanner for characterizing fuel element using film radiographic technique. This study is required to support the experimental work for the determination of burnup of irradiated fuel stored in the reactor pool of the RTP.

The overall concept, design, drawings, and circuit diagram were invented and developed by the main author

### Mechanical design

Radiographic techique is a simple method that can be used as a non-destructive analysis. This technique uses film to capture the radiation energy and the radioactivity is revealed by photographic emulsions [2]. This technique was used for environmental monitoring such as monitoring of radioactive fallout. The design of the scanner takes into account conditions in the reactor pool and safety matters to avoid any incident or accident during the experimental works. The spacing of the scanner, lifting process to move the collimator up and down and the loading and reloading of the film in the collimator compartment are among major design elements of the scanner.

There are two main type of drawings needed to be prepared before finalizing the conceptual design of the underwater radiography scanner. Firstly, three dimensional (3D) drawing was prepared to allow the designer to visualize the overall perspectives including components and the assembly design. Then, two dimensional (2D) drawings are prepared with much details including dimension to facilitate the subsequent fabrication process. All drawings are drawn using Inventor by Autodesk.

**Fig. 2:**
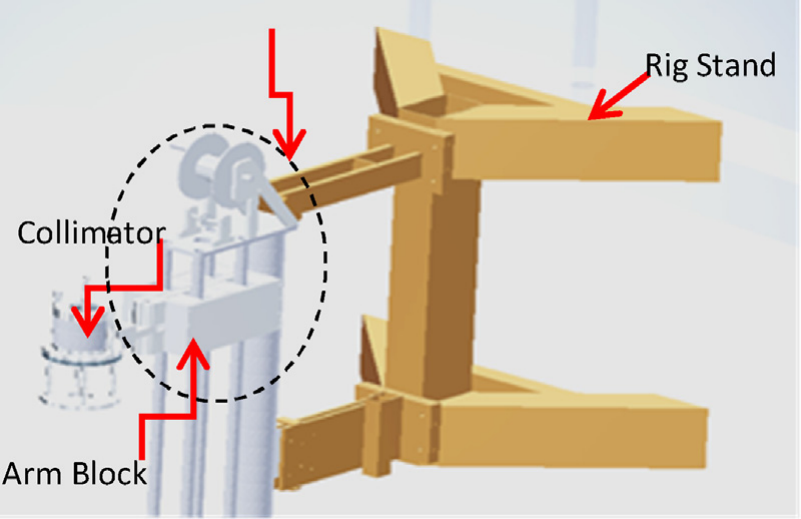
Conceptual drawing of the underwater radiography scanner in 3D.

When a design is performed in 3D, it assists designers with coordination. The designer can walk through a 3D model with specialized software and see the actual size and space of the design. [3]. It also allows the designer to see if their designs conflict with other disciplines or existing conditions they may not readily seen in 2D. For this particular application, the 3D design is superior as it can address safety concerns especially to see how much clearance and access around the surroundings in the reactor pool is available. Fig. 2 shows an example of the 3D conceptual drawing of the radiography scanner. This drawing depicts the main structure and component of the scanner which are the rig stand, arm block, collimator and lifting mechanism.

### Lifting mechanism

**Fig. 3:**
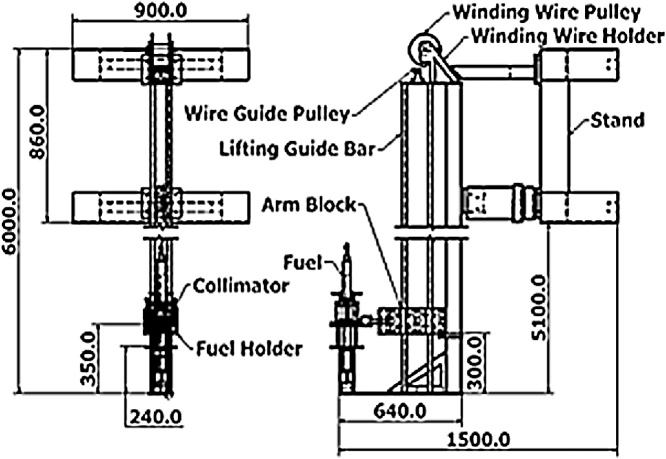
show 2D assembly drawing of the underwater radiography scanner.

For the 2D drawings, the designer needs to create several individual model and elevation view to see the exact space allocation [4]. Thus, the 2D views require multiple models to be developed and take longer to prepare. Fig. 3 shows the complete radiography scanner drawn in 2D view. On the right of Fig. 3 is the front view of the scanner where the mechanism to control collimator shutter is by means of pen cylinder. Next to it is the side view of the scanner. From this angle, illustration of a fuel element in the collimator is clearly pictured.

Lifting mechanism comprises of winding wire pulley, guide pulley, and guide bar. At the bottom of Fig. 3, cross section of the arm block is shown where the guide pulley modules are placed. These modules are used to retain the arm block in stable and correctly track position during the up and down lifting movement.

**Fig. 4:**
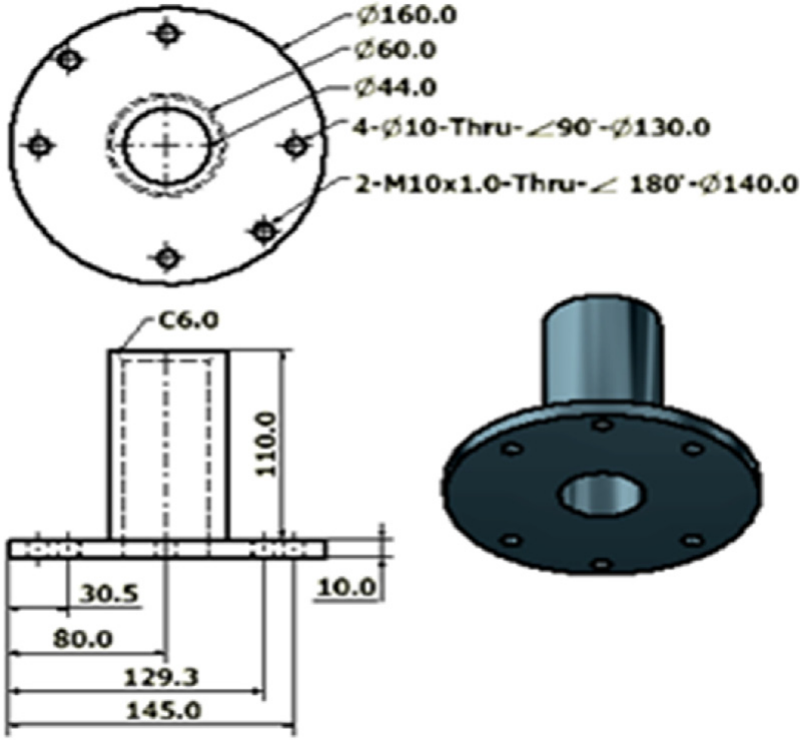
2D parts drawing of the shutter of the collimator.

Another purpose of 2D drawing is to construct fabrication drawings. In an assembly drawing, normally the dimensions of the final product such as height, width and length is depicted but in parts drawing, the dimension represents the actual components’ dimension during each fabrication steps such as machining, boring, welding and shaping towards achieving the final product specification. Fig. 4 is an example of parts drawing for the collimator shutter.

### Materials selection

Material selection is one of the foremost functions of effective engineering design as it determines the reliability of the design in terms of industrial and economical aspects, suiting the operational conditions such as loads, temperature, stress and strain. Hence, the following 5 elements were considered for the material selection inter alia mechanical properties, wear of materials, corrosion, and ability to manufacture and cost [5].

In this case, stainless steel 316, lead, aluminum 6061 and Teflon are the best materials selected to construct the underwater scanner.

### Stainless steel SX316

Since the reactor pool is sensitive to corrosion and scaling, stainless steel material is the option of material for bolt, nut, spring washer and washer, mainly use for fittings and assembling of the individual components of the scanner [6].

### Lead

Lead is a proven radiation shielding material especially for gamma radiation [7]. In this design, lead lining is furnished inside the collimator at its circumference wall, top lid and bottom base. The collimator shutter is fully fabricated from lead ingot through a molding process.

### Aluminum 6061

Aluminum 6061 is selected for the most part of the scanner structure except the rig stand which was fabricated using mild steel. Aluminum was chosen due to its lighter weight and corrosion resistant in water environment. Most importantly Aluminum exhibits less interaction with neutron radiation compared to stainless steel [8].

### Teflon

PTFE, also known under the brand name Teflon, is a soft fluoropolymer mechanical plastic with exceptional resistance to high temperatures, chemicals, corrosion and stress cracking. PTFE is also supreme against neutron attack [9], in addition to being water resistant and anti-scratch. Teflon material is chosen for guide bar pulley.

### Parts assembly

**Fig. 5:**
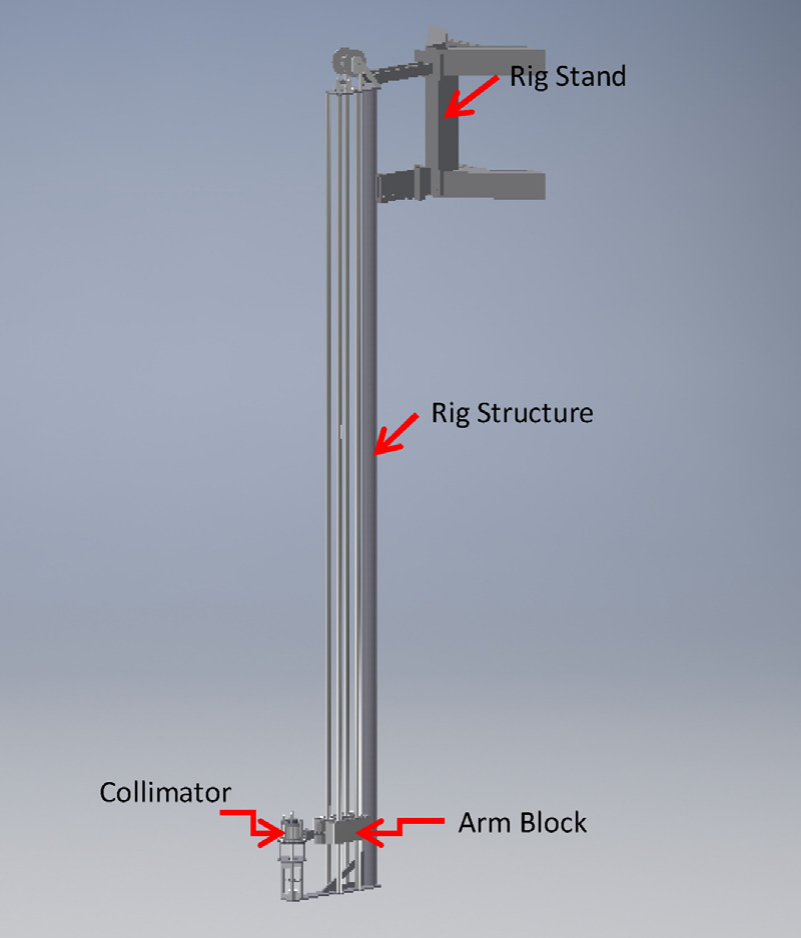
Perspective of full assembly of the underwater scanner rig in 3D view.

The underwater scanner consists of four main units which are the arm block, the collimator, the stand and the main structure. Fig. 5 shows the perspective of the underwater scanner in full assembly.

**Fig. 6:**
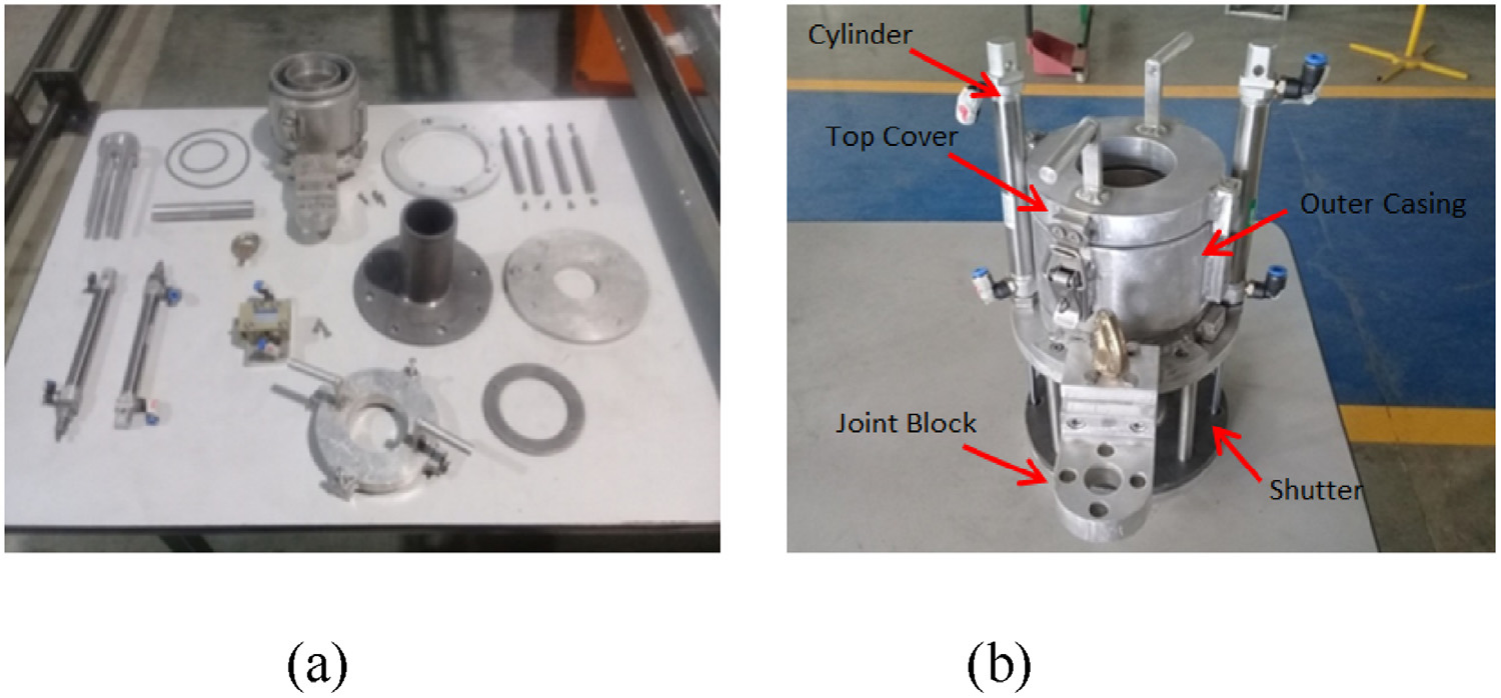
(a) Components of collimator and (b) the finished product.

Fig. 6(a) depicts the individual parts and the finished product of the collimator. It can be seen that attention to details assigned for each parts of this component in order to ensure the collimator fit the purpose. In particular, refer to Fig. 6(b) the finished product was represented the collimator condition that shall be perfectly water-tight, provide adequate shielding and able to control the radiation exposures. The collimator underwent several testing and quality check primarily to avoid leakage.

Overall, the underwater radiography scanner took one year to construct from the design stage to fabrication to component testing and finally full assembly.

### Control system

There are two control systems used in the scanner namely the electro-pneumatic system and the lifting system.

### Electro-pneumatic system

**Fig. 7:**
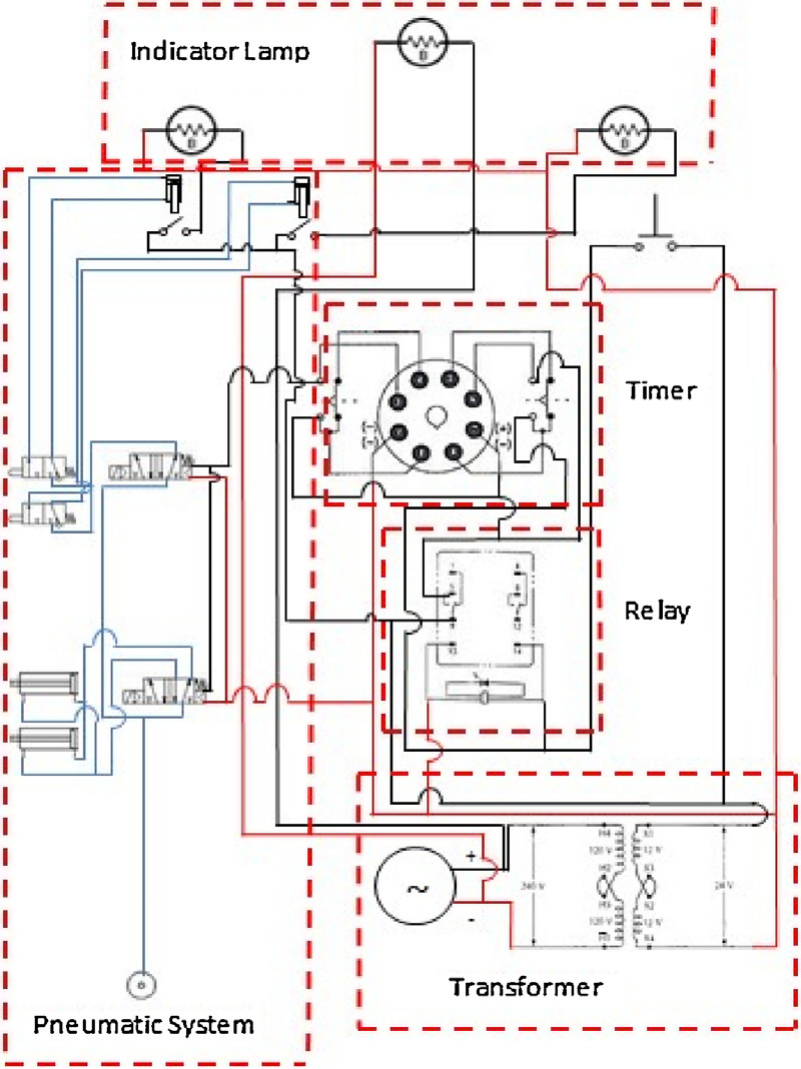
Circuit diagram of the electro-pneumatic system.

This system is created to control up and down movement of the shutter located inside of the collimator. This system is equipped with timer to control the exposure time. The timer is set at 300 s and the air pressure used is 50 psi. Beside the timer, relay is installed to program the sequence for controlling the actuator. Step down transformer is used to reduce incoming power from 240 V to 12 V in alternative current (AC). Another novel feature is the underwater sensor which was created using micro actuator. This sensor is a safety measure to indicate through indicator lamp as whether the shutter has been opened or closed during the operation. The circuit described above is illustrated in Fig. 7.

### Lifting system

**Fig. 8:**
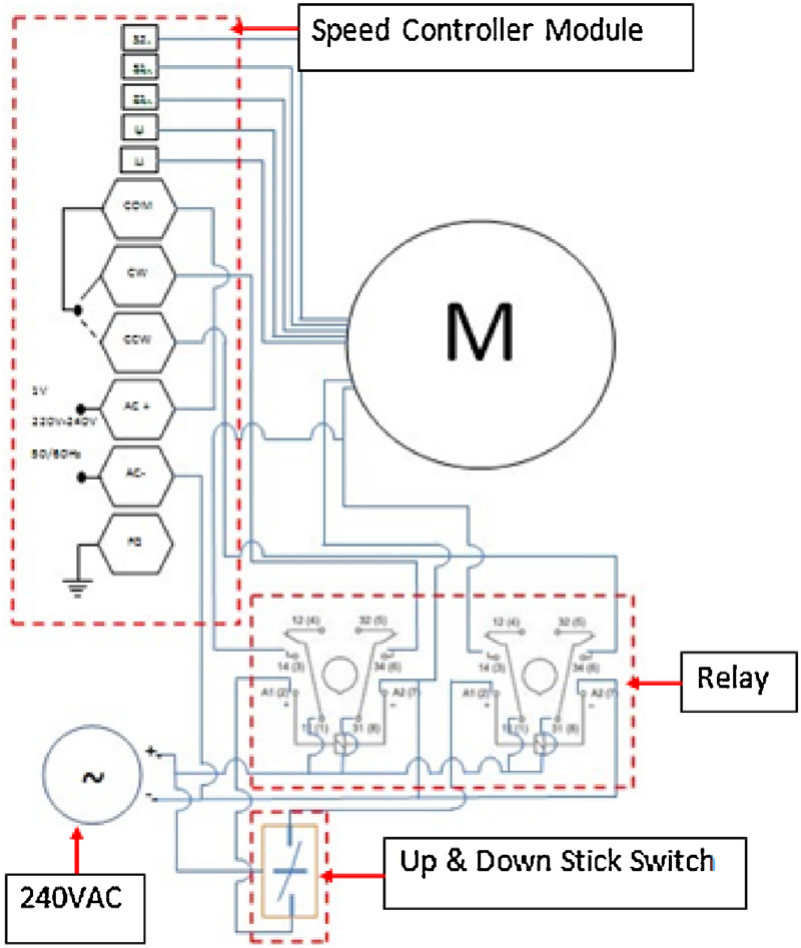
Circuit diagram of the lifting system.

Lifting system is built to control up and down movement of the collimator in the reactor pool. The system is equipped with 240 VAC stepping motor with initial tork of 148 kgs/cm and gear head with ratio 1:30. The motor rotation is controlled by a speed controller. The circuit diagram of the lifting system is depicted in Fig. 8.

### Installation

**Fig. 9:**
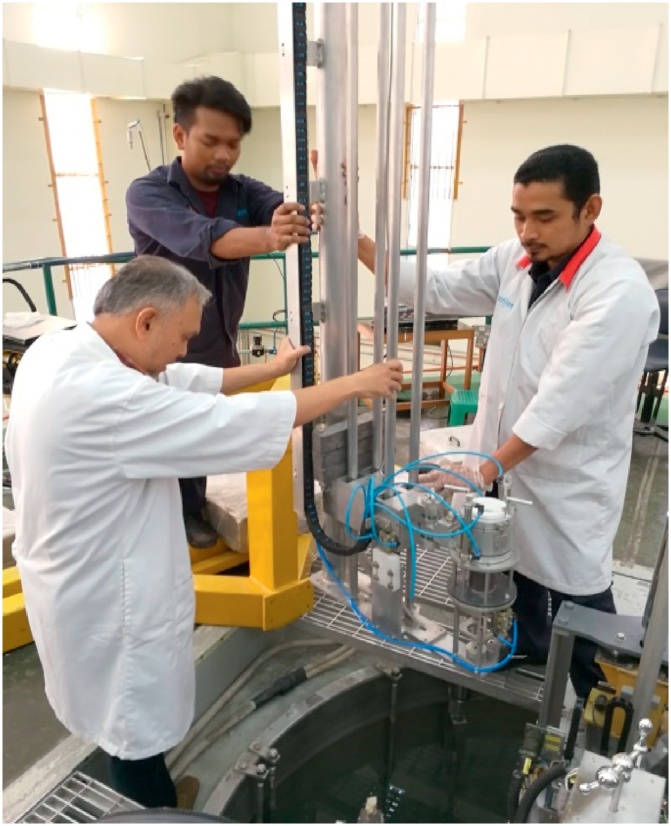
Rig is carefully aligned before lowering it into the pool.

**Fig. 10:**
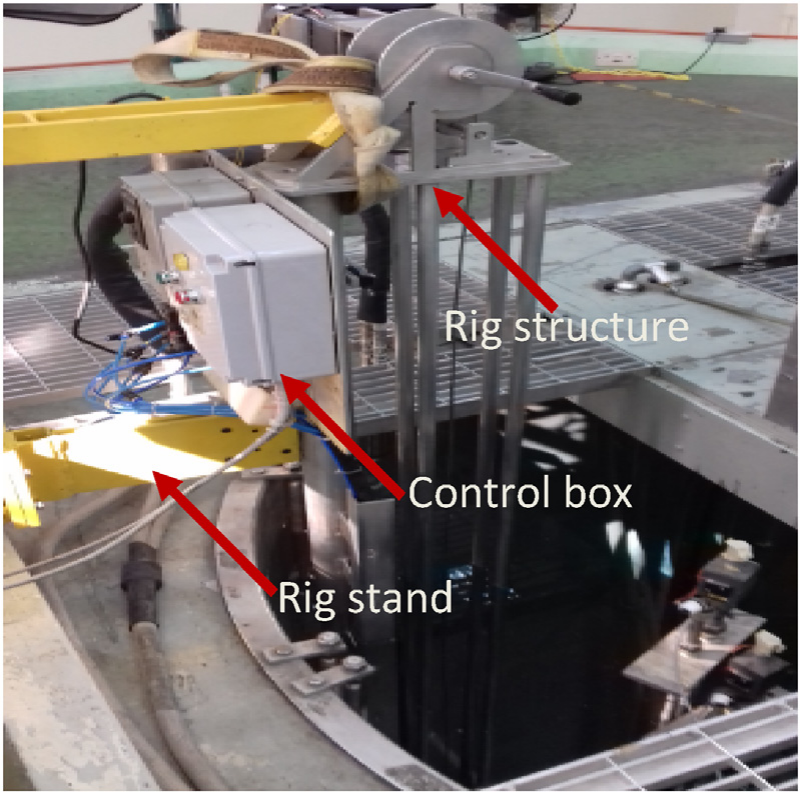
The final position of the underwater scanner.

**Fig. 11:**
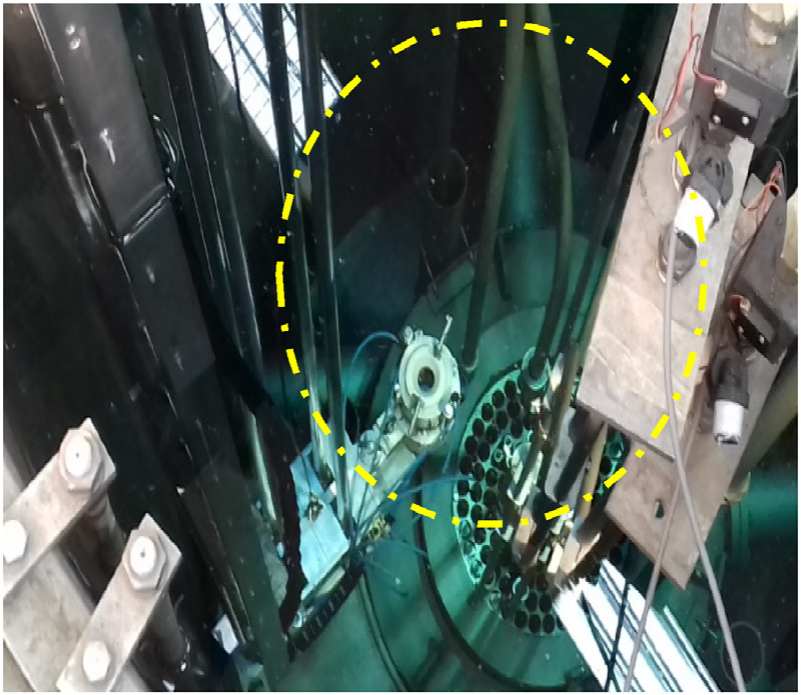
Position of the underwater scanner in the pool (in circle).

To securely install the scanner, the stand is fixed at the top of reactor pool, while the rig structure is submerged 5-meter-deep into the reactor pool. Lowering the structure is a critical step and requires careful alignment so that the structure would not disturb and get contacted with any of the existing components and piping in the pool. Fig. 9 was taken during the installation stage and Fig. 10 shows the final position of the scanner in the top of reactor platform while Fig. 11 shows the scanner in underwater at RTP pool upon completion of the installation.

### Testing and validation of the radiography scanner

The most important aspect before the scanner can be used for experimental works is testing and validation. Two types of testing were carried out; dry run test and wet run test. In dry run test, the scanner is examined for stability and operational performance. Dry run test is carried out in controlled conditions such that negative effect from any functional failure is minimized. In wet run test, the operational performance in real environment is evaluated where in this case, inside the reactor pool tank. All sub-systems were examined and operating procedures for the scanner were developed accordingly.

**Fig. 12:**
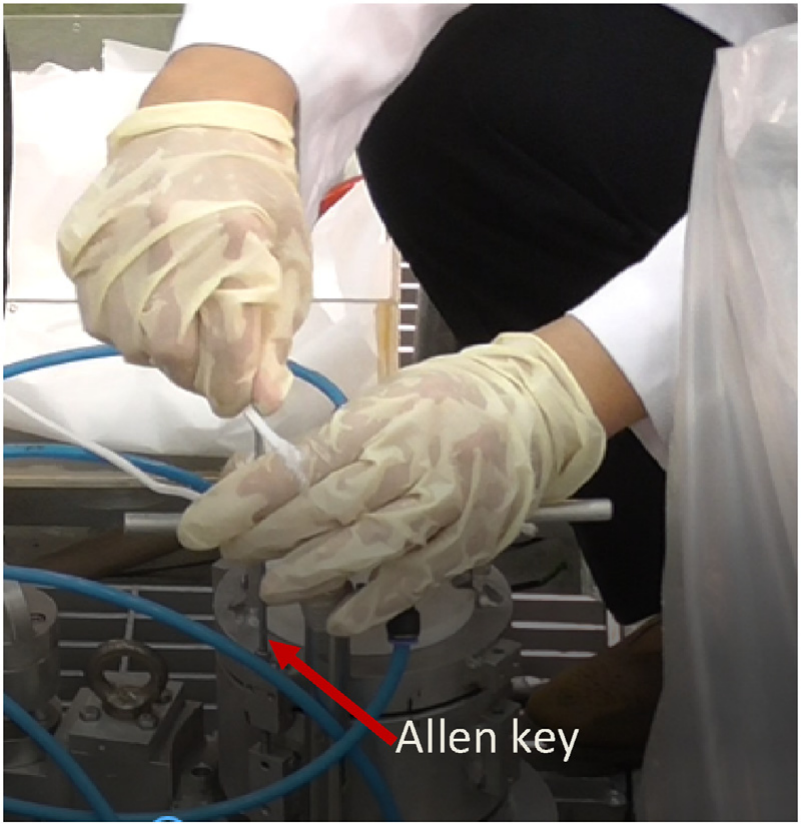
The collimator cap is unscrewed to open using an Allen key.

In terms of preparing the film sample for testing, it is imperative to follow the sequence rigorously to ensure water does not infiltrate due to water pressure when the scanner is at 5 m deep in the reactor pool. Water molecules stained the film and compromised the radiography image altogether. The collimator cap is tightened with 4 Allen cap bolts, which need to be removed using Allen key before the film can be inserted into the compartment of the collimator. Fig. 12 shows how the cap is unscrewed to open the collimator cap using an Allen key.

**Fig. 13:**
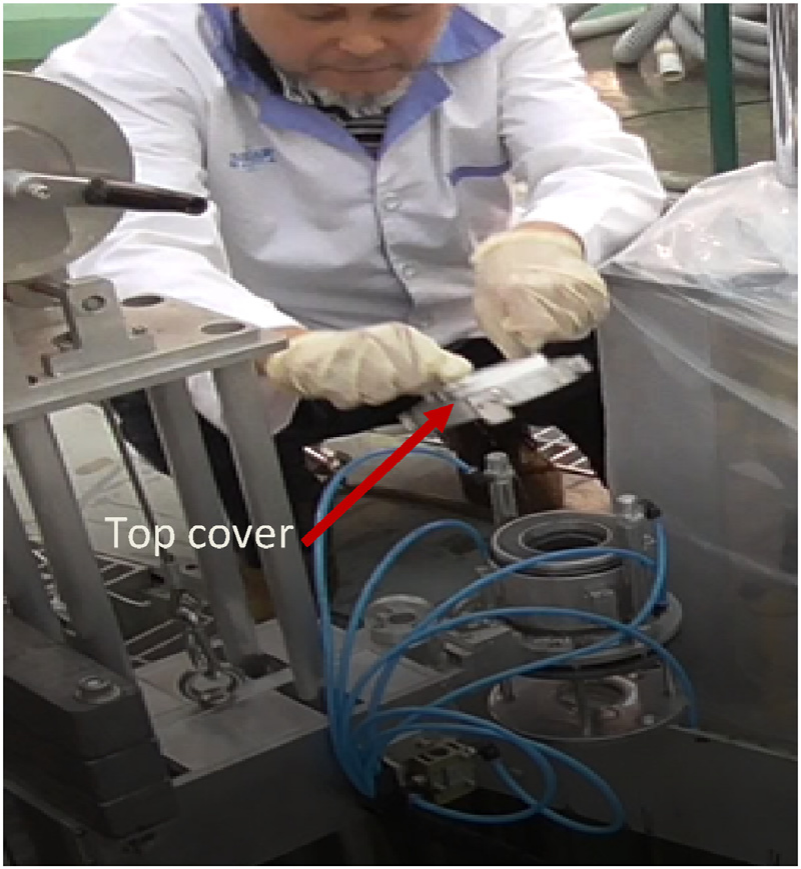
Collimator cap is unscrewed and removed.

**Fig. 14:**
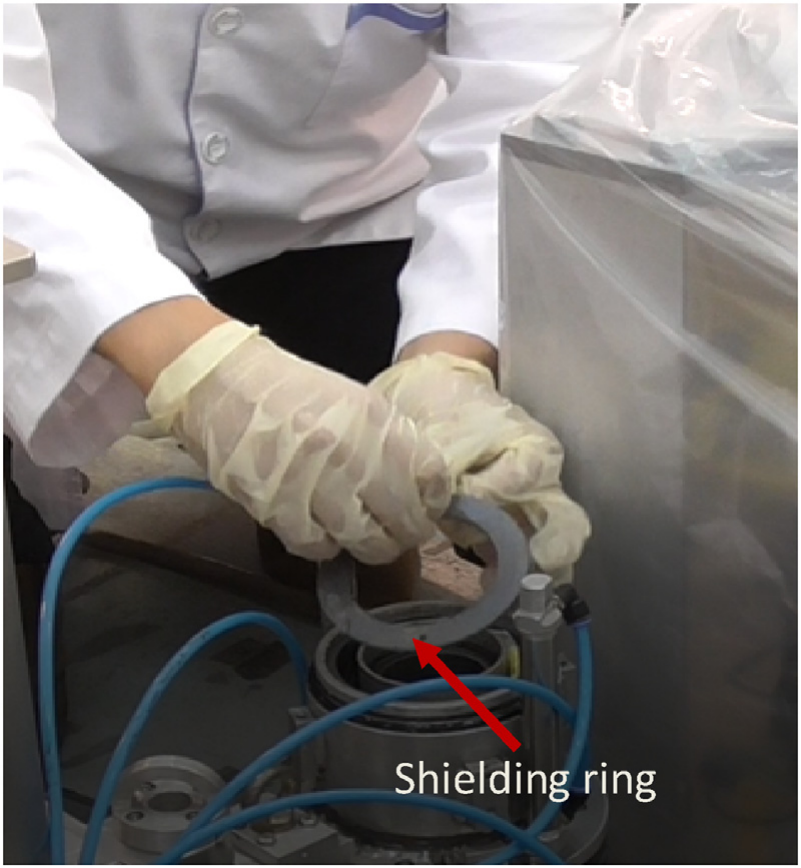
The shielding ring is removed to get access to the film compartment.

**Fig. 15:**
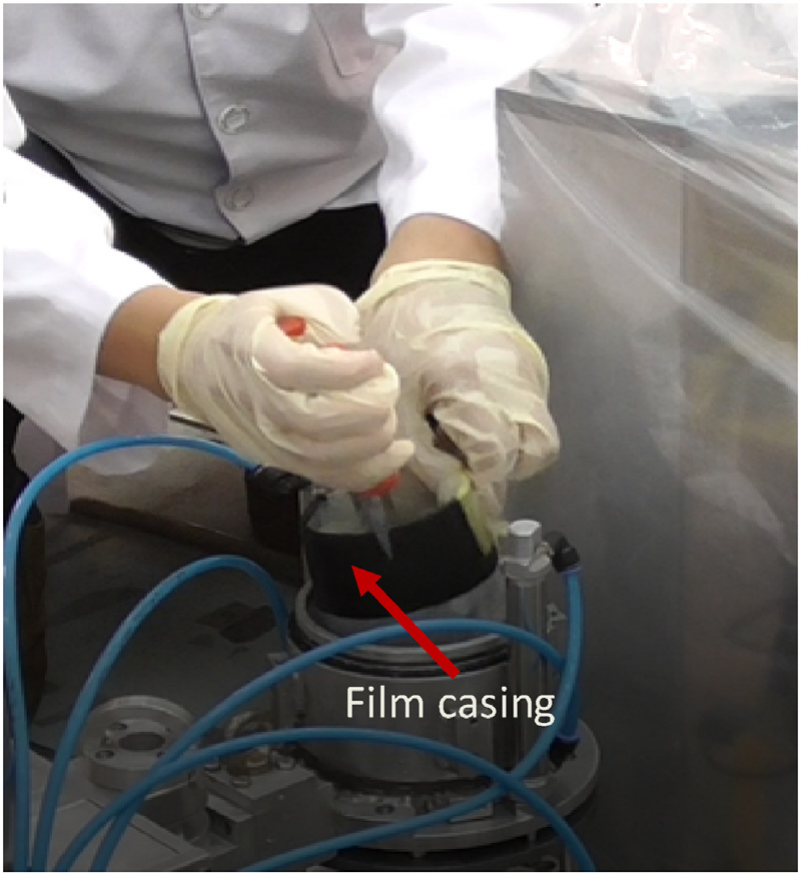
Film is inserted into the compartment.

**Fig. 16:**
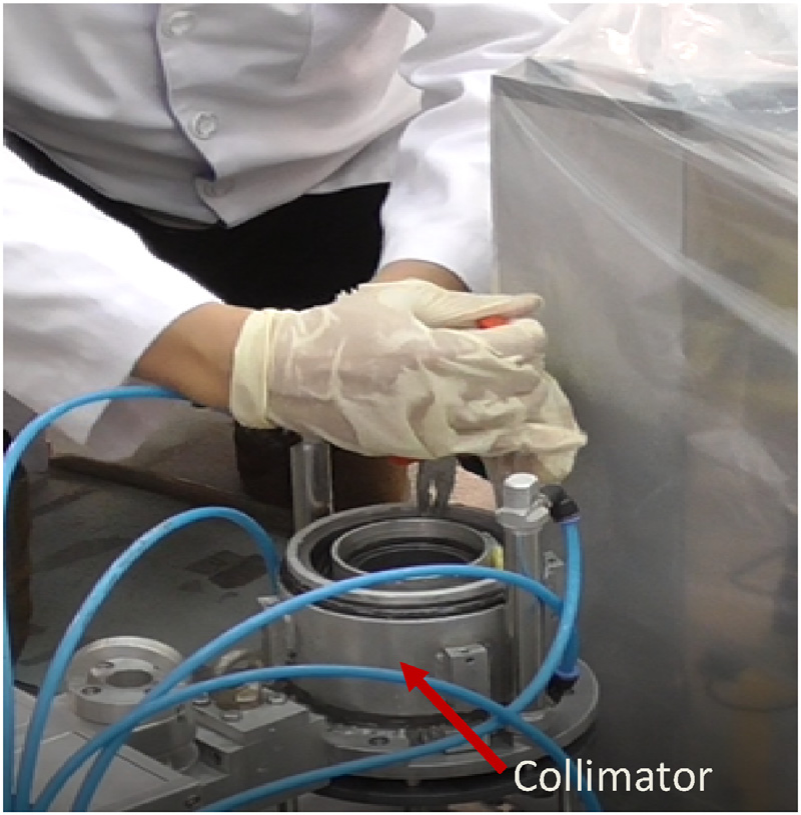
It is important to ensure the film is properly position inside the compactment.

When the cap is opened, a shielding ring that is fabricated from lead is seen (Fig. 13). This ring is removed and one can see the compartment of which the film will be inserted (Fig. 14).Then, the film is inserted into the compartment properly (Fig. 15). A little pressure may need to be applied to slot the film in (Fig. 16), as the compartment is measured to fit one film tightly. The length of the film is previously measured to match the compartment length so that the film is not overlapping.

**Fig. 17:**
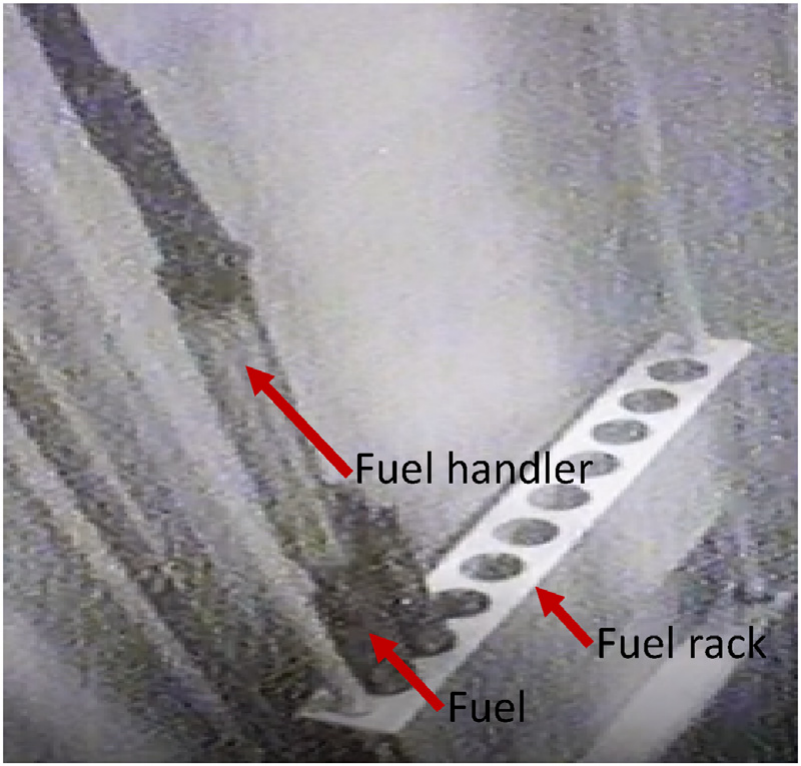
The fuel handler is used to move a fuel from a storage rack.

After the film has been fixed inside the compartment, the shielding ring is placed and the cap is secured tightly. Then, the collimator is ready to be lowered down into the pool. Once the scanner is in its position, transfer of the selected irradiated fuel is initiated. This operation is made possible with the use of fuel handler that grip and release the fuel using mechanical force. A high resolution (1080 pixel) underwater camera is used to assist the fuel movement as one of the cautionary measures when operating in the sensitive reactor pool environment. Fig. 17 shows the fuel handler is used to move a fuel from a storage rack.

**Fig. 18:**
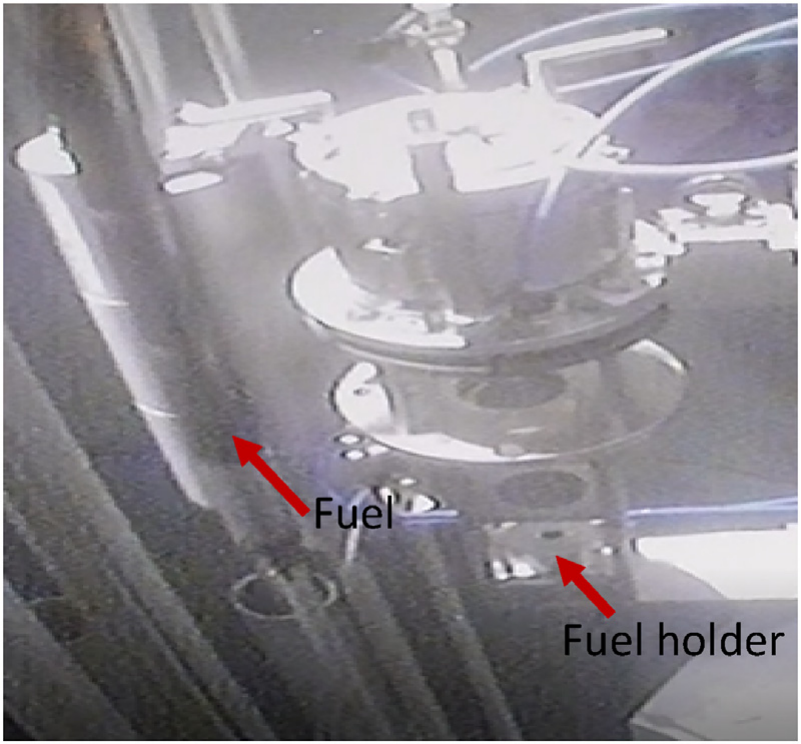
A selected fuel is brought to the collimator and put in at fuel holder.

**Fig. 19:**
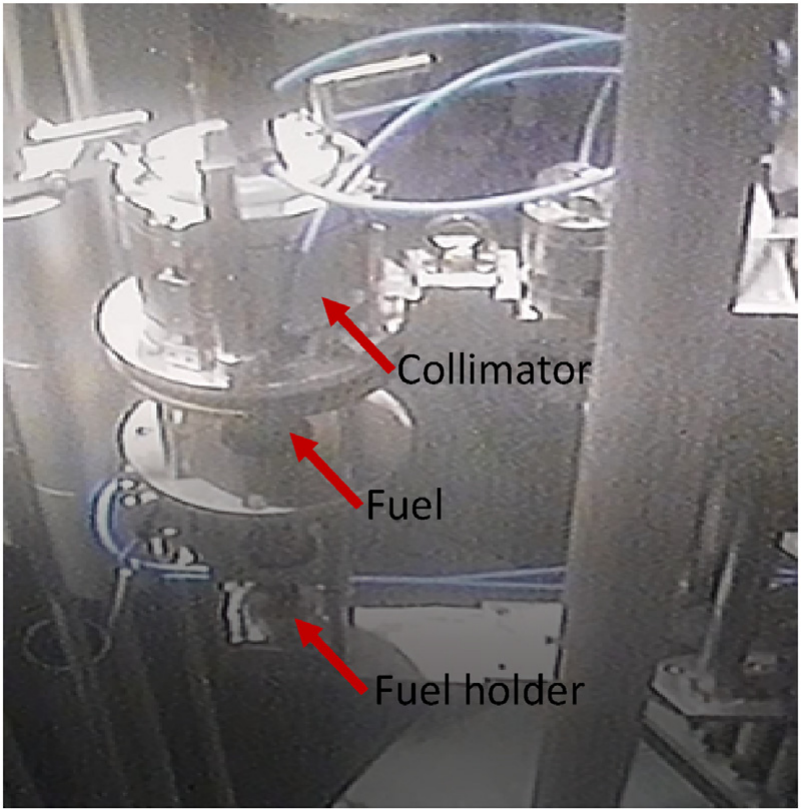
Image of the fuel successfully positioned in the fuel holder ready for testing.

In this work, a fuel located in the storage rack is selected for the radiography analysis. The fuel is carried and brought near to the collimator, and then slowly the fuel is inserted into the center hole of the collimator until it sits completely in the fuel holder as shown in Fig. 18, Fig. 19. Once in this position, the set up for the testing is complete and may begin.

To run the test, the scanner will be operated following the sequence dictated as follows:

• Rig is positioned and secured, and every sub-system is checked
• Switch ‘ON’ the system. Light indicator will flash, indicating the system is ready to ‘RUN’.
• At the top of the rig, film is inserted into the collimator slot and secure properly.
• Using lower switch, the arm block where collimator is attached to, is lowered down to the scanner platform, located right at the end of the scanner, when the arm block touches the platform, green light indicator turned ON.
• Irradiated fuel selected for the test is transferred into the fuel holder.
• Once the fuel is in position, the collimator shutter is opened and the timer is automatically switched ‘ON’. Set the timer as intended.
• When the set time is over, the shutter will retract to its original position and the collimator window is closed subsequently. Red light turned ‘OFF’.
• Flick the upper switch. The arm block is carried upwards via the lifting mechanism to the top of the reactor. Green light turned ‘OFF’.
• Collimator cap is removed and the exposed film is retrieved for film processing.

The type of film used is AFGA STRUCTURIX D4 NIF cut to size 8.0 cm x 25.4 cm. The control sample is obtained from film being exposed to the fuel with shutter in closed position. Films were developed using the standard film development procedures in the dark room. Next, the image is digitized using CIT Film Digitizer Model DR3000 HD with display resolution 2560 × 2048 (5 MG) pixels. Further on, the digital image is analyzed using Image J software where the black spots of the film are translated into pixels to correspond the correlation between gamma energy and the darkness of the film image.

### Testing and validation result

The main output from the experimental works conducted using the underwater radiography scanner is the radiography film image where this image is developed when the film is exposed to gamma radiation from an irradiated fuel. Even a small water leakage into the film will compromise and probably damage the film. It was observed that the scanner is working perfectly as per design in the underwater environment that exerts pressure around 150Kpa. No water leakage is detected inside the collimator compartment, while the lifting movement of the arm block was smooth and stable.

**Fig. 20:**
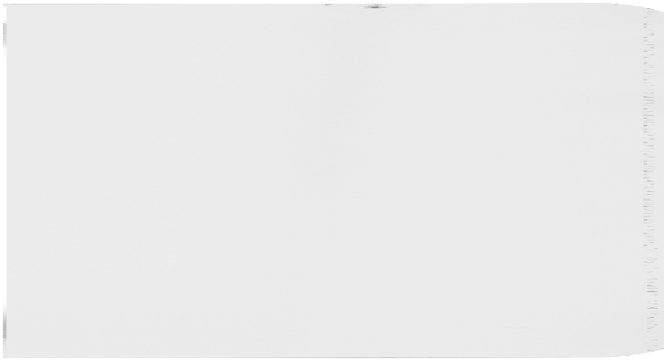
Radiography film image when the film is exposed to the 12.0 wt% of nominal uranium-235 fuel when the shutter is closed.

**Fig. 21:**
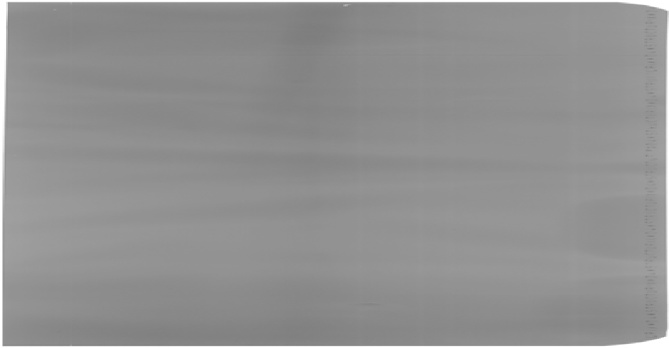
Film image observed from exposure to the 8.5 wt% fuel. Black spots indicating presence of silver on the film.

**Fig. 22:**
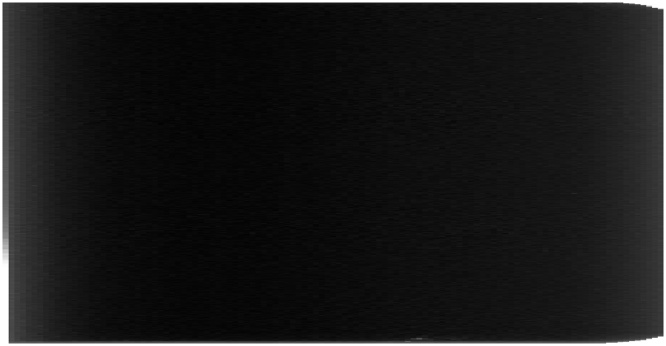
Gamma energy from 12.0 wt% of nominal uranium-235 fuel captured by film in opened-shutter position.

The film image would not appear until the film has been developed. The associated control film image is shown in Fig. 20. It can be deduced from Fig. 21 that for control sample, there was very limited interaction between gamma radiation and AgBr in the film hence the AgBr became inactive as compared to the interaction between film and gamma radiation when the shutter was opened. Fig. 22 shows an example of the film image when the film captured gamma energy through the exposure to an irradiated fuel. Each interaction between film and gamma radiation will have imparted a black spot of activated AgBr as the compound interacts with hydro quinine (in developer composition) during the film processing to reduce to silver atom. These black spots are then amplified by chemicals to the film negative.

Theoretically, gamma energy radiating from a fuel increases with increasing quantity of uranium composition in the fuel. Therefore, the degree of gamma exposure and the subsequent gamma-film interaction increases when the film is exposed to fuel with 12 wt% uranium compared to the film exposed to 8.5 wt% uranium-235. Fig. 22 shows the film when exposed to the 12 wt% uranium-235.

Film image is digitized for further analysis. Digital images are a made up of a matrix of energy intensity points called pixels [10]. The most common pixel format is the byte image, where this number is stored as an 8-bit integer giving a range of possible values from 0 to 255. In more advanced systems, 2^12^ gray values (0–4095), 2^14^ or 2^16^ gray levels are used [11]. The brightness of the pixel is represented by its gray value. Typically, zero gray value is regarded as black while gray value of 255 is regarded as white [12].

**Fig. 23:**
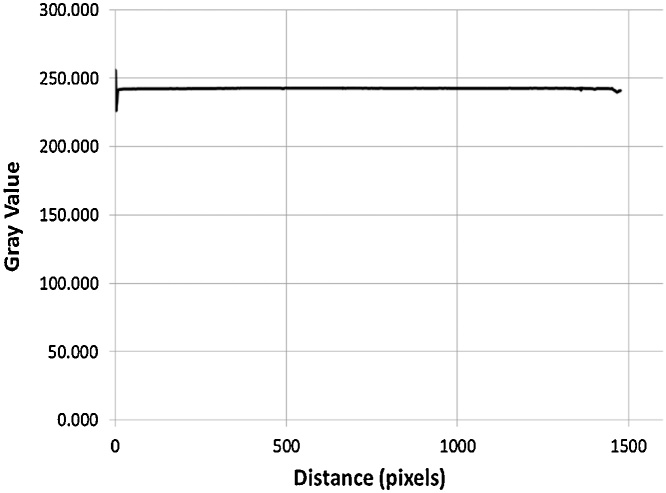
Gray value of the control sample.

The result shown in Fig. 23 showed that the control sample has a gray value of 242 implying that this number is highest gray value would be obtained from the experimental works, attributed to the combined effect of the least radiation or gamma energy captured (shutter in closed position) and the film development process in the dark room.

**Fig. 24:**
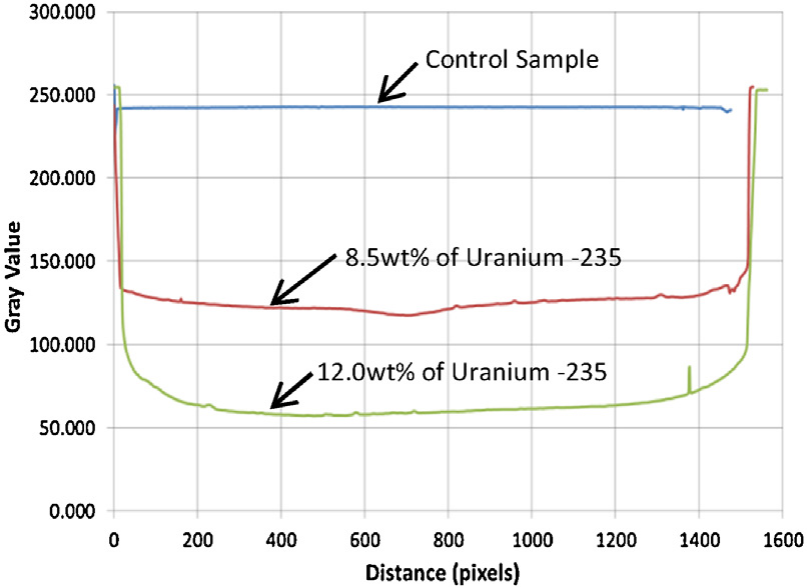
Gray values for control sample and the film exposed to the 8.5 wt% fuel and the 12 wt% fuel.

The gray value decreased drastically by 53% from 242 to 128 when the film was exposed to 8.5 wt% U-235 fuel. The gray value further dropped when the film was exposed to 12 wt% fuel, from 128 to 59, in consistent with the film which has become darker. Fig. 24 summarizes the trend of the gray value for the control sample, and the film exposed to 8.5 wt% and 12.0 wt% uranium-235 respectively. When compared to the control sample, the gray value for film exposed to 8 wt% and 12 wt% fuel is 52.8% and 75.6% respectively.

**Fig. 25:**
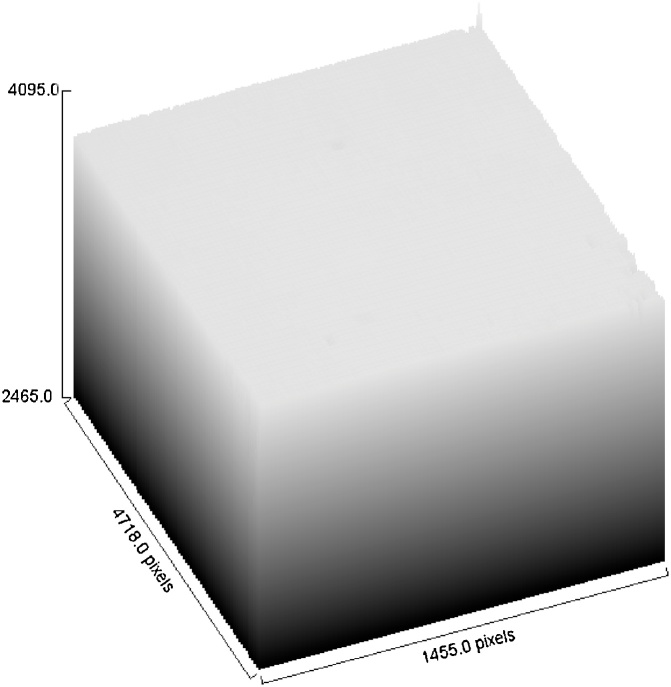
3D view of pixels for the control sample. Shutter is in close position.

**Fig. 26:**
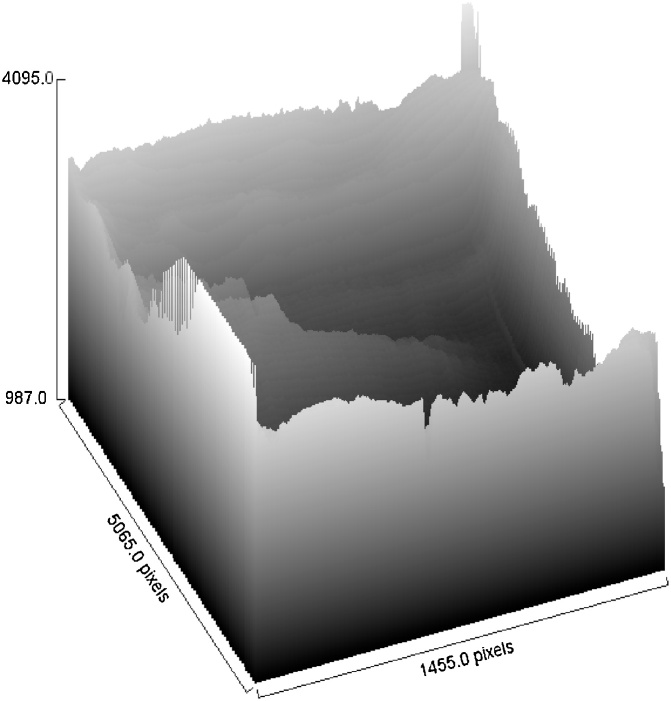
3D view pixels for film exposed to 8.5 wt% uranium fuel.

**Fig. 27:**
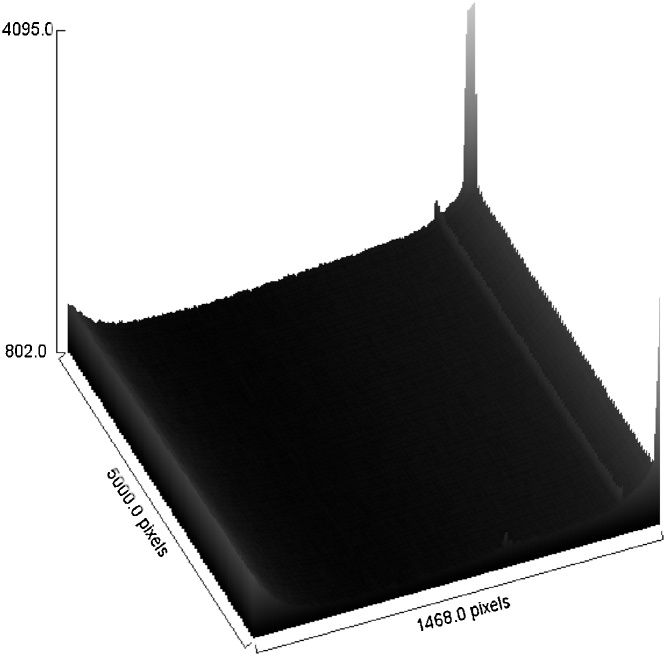
3D view pixels for film exposed to 12 wt% uranium fuel.

Further on, observation of the pixels from a 3D view allows analysis of the degree of gamma-film interaction, in particular the amount of gamma energy emitted from the fuel as well as the degree of radiation energy captured by the film. The energy produced is depending on fission reaction and also depending to weight percentage of uranium-235 content. More fission of uranium content leads to higher energy produced [13] and subsequently less brightness of the pixels. An example of 3D view of the pixels is given in Fig. 25, Fig. 26, Fig. 27.

Photographic films are coated with silver bromide (AgBr) crystals [14] which become activated when gamma energy in the form of photons hit the compound. Through the film development process that involves a series of oxidation/reduction reactions, the activated AgBr is ultimately reduces to elemental silver. The amount of silver ions deposited in the film is directly proportional to the amount of gamma energy hitting a given region of the film.

Fig. 25 depicting the 3D view pixel from the control sample shows that no interaction between gamma radiation and the film exposed. In this situation the black and white development is the fixation to removes the remaining inactivated AgBr to give clear and transparence regions in the photographic negative but in Fig. 26 showing analysis of the pixel for film exposed to 8.5 wt% uranium fuel indicates that a significant portion of AgBr has been removed from the film and reduces as silver deposit on the film.

The changing 3D view profile for the pixel analyzed for film exposed to 12 wt% uranium fuel further characterizing how seriously the photons have hit the film. Refer to Fig. 27 3D view pixels for film exposed to 12 wt% uranium fuel. Most of the AgBr coating has been reduced to elemental silver when the film is exposed to the higher radiation from the fuel with higher uranium-235 content. The pixel properties were reduced markedly by 29.3% when compared to the control sample making the film darkest of all the samples analyzed.

## Conclusion

An underwater radiography scanner is designed and developed in this work as a means of fuel characterization study. The main design elements incorporated include dimensions, space consumption, water tightness, operation underwater approximately at 5 m deep and control of radiation exposure. To assists during the designing stage, engineering drawings were prepared in 2D and 3D view. The materials used to construct the scanner are aluminum 6061, lead and Teflon. Three main units that make up the scanner are main structure, arm block for lifting mechanism and collimator. The scanner is equipped with electro-pneumatic control system and lifting system. Lowering the scanner into the pool is a critical step to avoid violating safety of the reactor. All systems and sub-systems underwent dry test before full assembly and wet testing was performed. Radiation in terms of gamma energy is captured by radiography film which after development and processing revealed the image of the radiation impact in terms of pixel and gray value. It was found that the experimental outputs obtained from utilizing the scanner are valid and further confirms that the scanner is ready to be used for fuel characterization study.

## References

[1] Alnour I.A., Wagiran H., Ibrahim N., Hamzah S., Wee B.S., Elias M.S., Karim J.A. (2013). Determination of neutron ﬂux parameters in PUSPATI TRIGA mark II research reactor. Malays. J. Radioanal. Nucl. Chem..

[2] Tochilin E., Shumway B.W., Kohler C.D. (1956). Response of photographic emulsions to charged particles and neutrons. J. Radiat. Res. Soc..

[3] Jackson T.R., Liu H., Patrikalakis N.M., Sachs E.M., Cima M.J. (1999). Modeling and designing functionally graded material components for fabrication with local composition control. J. Mater. Des..

[4] Papamichael K., Chauvet H., LaPorta J., Dandridge R. (1999). Product modeling for computer-aided decision-making. Autom. Constr.: An Int. Res. J..

[5] Ljungberg Lennart Y. (2007). Materials selection and design for development of sustainable products. J. Mater. Des..

[6] Goudarzi N., Peikarim M., Reza Zahiri M., Reza Mousavi H. (2012). Adsorption and corrosion inhibition behavior of stainless steel 316 by aliphatic amine compounds in acidic solution. J. Inst. Metall. Mater. Sci. Committee Metall. Pol. Acad. Sci..

[7] King J.N., Champlin A.M., Kelsey C.A., Tripp D.A. (2002). Using a sterile disposable protective surgical drape for reduction of radiation exposure to interventionalists. Am. J. Roentgenol..

[8] Monnet I., Dubuisson P., Serruys Y., Ruault M.O., Kaı¨tasov O., Jouffrey B. (2004). Microstructural investigation of the stability under irradiation of oxide dispersion strengthened ferritic steels. J. Nucl. Mater..

[9] Kemmish D.J. (2011). Practical Guide to High Performance Engineering Plastics.

[10] Petrou M., Petrou C. (2010). Image Processing: The Fundamentals.

[11] Grahn H.F., Geladi P. (2007). Technique and Application of Hyperspectral Image Analysis.

[12] Kumar T., Verma K. (2010). A theory based on conversion of RGB image to gray image. Int. J. Comput. Appl..

[13] Bohr N., Wheeler J.A. (1939). The mechanism of nuclear fission. Phys. Rev. J..

[14] Marchetti A.P., Muenter A.A., Baetzold R.C., McCleary R.T. (1998). Formation and spectroscopic manifestation of silver clusters on silver bromide surfaces. J. Phys. Chem. B.

